# MDER-MA: A multimodal dataset for emotion recognition in low-resource Moroccan Arabic language

**DOI:** 10.1016/j.dib.2025.112005

**Published:** 2025-08-25

**Authors:** Soufiyan Ouali, Said El Garouani

**Affiliations:** Faculty of Sciences Dhar El Mahraz (FSDM), Sidi Mohamed Ben Abdellah University, LISAC laboratory, Fez, 30000, Morocco

**Keywords:** Emotion recognition, Moroccan Arabic, Darija, Low-resource language, Arabic dialects

## Abstract

Emotion recognition and analysis have become increasingly important in recent years, particularly with the rapid digitization and automation of virtual agents. As these systems are integrated into various aspects of daily life, enabling them to understand and respond to human emotions is essential for creating more natural, empathetic, and effective interactions. Humanizing virtual agents through emotion-aware capabilities enhances user experience, plays a critical role in emotion-driven services, such as personalized customer support and mental health assistance, thereby offering significant socio-economic benefits. Despite the significant advancements achieved in high-resource languages within this field, these results cannot be directly generalized to low-resource languages such as Moroccan Arabic. This is because emotional expression is highly influenced by cultural, regional, and linguistic factors, leaving a gap between research on high- and low-resource languages. One of the major challenges hindering the development of such a powered system is the lack of a high-quality and realistic dataset. This paper introduces MDER-MA, a comprehensive multimodal dataset designed for emotion recognition in the Moroccan Arabic dialect. Contains 5288 data items that express one of the four emotions: Happy, Sad, Angry, and Neutral, expressed in four different modalities: audio, text, spectrogram, and Mel-spectrogram images. Each modality contains 1322 samples. The samples were collected from various regions across Morocco to ensure the creation of a representative dataset that is not biased toward any single geographic or linguistic area. MDER-MA supports multiple applications, including emotion recognition, audio transcription, age and gender identification from both speech, text, and image modalities. Annotation was conducted by five native Moroccan speakers, ensuring high linguistic reliability for real-time emotion recognition tasks. This work aims to bridge the gap between high-resource and low-resource languages in the field of emotion-aware and humanized intelligent systems, and to foster the development of Arabic language technologies, with particular attention to regional dialects such as Moroccan Arabic.

Specifications Table

**Table:** 

Subject	Computer Sciences
Specific subject area	Emotion recognition, Artificial intelligence, emotion analysis in the Moroccan dialects.
Type of data	Image, Audio, Text, Raw
Data collection	Data were collected from reality TV shows, radio programs, podcasts, and interviews expressing one of four emotions: happy, sad, angry, or neutral. Audio is segmented into short clips using Audacity. Only clips with clearly expressed emotions were retained after manual validation. Spectrograms and Mel-spectrograms were generated using Python. All audios were transcribed manually by native Moroccan Arabic speakers.
Data source location	Faculty of Sciences Dhar El Mahraz (FSDM), Sidi Mohamed Ben Abdellah University, LISAC laboratory,​ Fez​, Morocco, 30,000.
Data accessibility	Repository name: Mendeley Data: ERD-MA: Emotion Recognition Dataset for the Moroccan Arabic Data identification number: 10.17632/yzsw3ff6rn.1 Direct URL to data: https://data.mendeley.com/datasets/yzsw3ff6rn/1
Related research article	None

## 1 Value of the Data

• **First Emotion Dataset for Moroccan Arabic:** This is the first multimodal emotion recognition dataset specifically designed for Moroccan Arabic, a low-resource dialect. It fills a significant gap in the availability of emotion-labeled resources for North African Arabic varieties.
• **Multimodal Design for Versatile Use:** The dataset includes audio, text transcriptions, spectrograms, and Mel-spectrograms, making it suitable for multimodal emotion recognition in Darija and automatic speech recognition.
• **High-Quality Annotations by Native Speakers:** All audio annotation and transcriptions were conducted by five native Moroccan Arabic speakers, ensuring linguistic accuracy and contextual understanding. This adds to the dataset's authenticity and makes it highly reliable for both academic and industrial research.
• **Culturally Relevant Emotional Expression:** Emotional expression varies greatly across cultures and regions. This dataset captures authentic Moroccan emotional expressions across all Moroccan regions, making it valuable for research in cross-cultural emotion modeling and culturally aware AI systems.

## 2 Background

Moroccan Arabic (Darija) is a widely spoken dialect, used daily by over 38 million people [1]. Despite its widespread use, it remains severely underrepresented in computational linguistics and lacks the linguistic resources necessary for advancing research in speech and emotion processing. This dataset was compiled to address this gap by providing a multimodal resource specifically designed for emotion recognition in Moroccan Arabic.

Although numerous datasets have been developed for emotion recognition in Arabic, most are either in Modern Standard Arabic (MSA) or other regional dialects. For instance, in [2], the authors introduced the KEDAS dataset, which consists of 5000 audio recordings from 500 actors expressing five emotions (anger, happiness, sadness, fear, and neutrality). Similarly, the BAVED dataset [3] provides 1935 audio samples of isolated Arabic words recorded by 61 speakers and labeled across three emotional intensity levels. Another example is the King Saud University (KSU) dataset [4], which contains approximately five hours of MSA emotional speech from 23 speakers, covering six emotion categories. For dialectal resources, a Saudi dialect dataset was introduced in [5], including 175 recordings across four emotion labels, while the EYASE database [6] offers 579 utterances in Egyptian Arabic across angry, happy, neutral, and sad emotions. Despite these valuable contributions, datasets in MSA or other dialects cannot be directly generalized to Moroccan Darija, as it differs significantly at the phonological, lexical, and syntactic levels. Darija is characterized by a rich blend of Amazigh, Arabic, French, and Spanish influences, which makes it linguistically distinct from both MSA and other Arabic dialects. Consequently, models trained on these existing datasets often fail to capture the unique prosodic, cultural, and emotional cues embedded in Moroccan speech.

The motivation for creating this dataset stems from the growing need for human-centered AI systems that can interpret and respond to users' emotions, particularly in languages and dialects that have traditionally been overlooked. While emotion recognition has progressed considerably in high-resource languages, such progress is limited for low-resource dialects like Darija. The dataset was developed using authentic audiovisual content to complement ongoing research on emotion-aware systems for Moroccan Arabic and offers the broader research community a reproducible, culturally grounded dataset that can serve as a foundation for further exploration.

## 3 Data Description

**Fig. 1:**
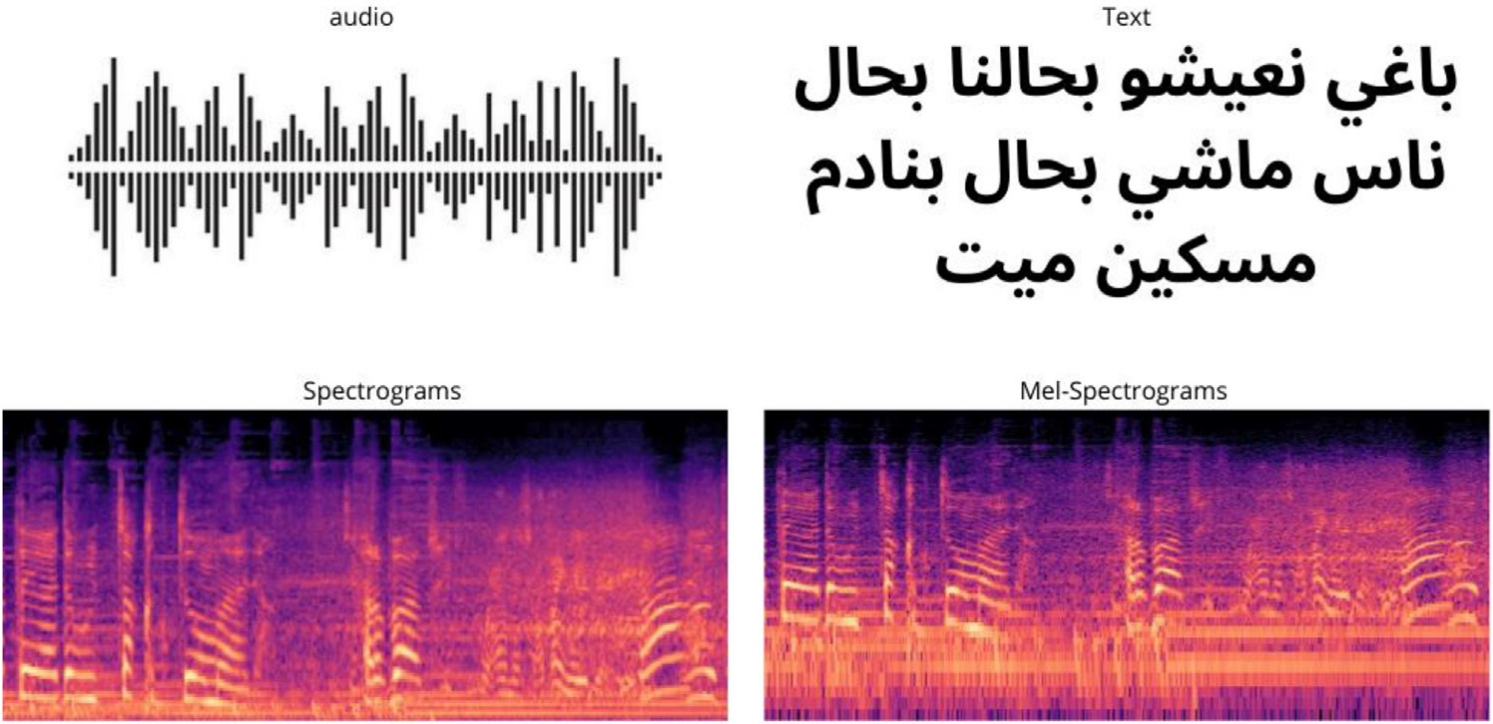
MDER-MA different modalities.

The MDER-MA, Multimodal Emotion Recognition dataset, contains 5288 data items that express one of the four emotions: Happy, Sad, Angry, and Neutral, expressed in four different modalities (as shown in Fig. 1) audio, text, spectrogram, and Mel-spectrogram images. Each modality contains 1322 samples. The audio data were manually collected and annotated to ensure the inclusion of only clips that clearly and directly express one of the selected emotions. Similarly, the text data were manually transcribed by native Moroccan Arabic speakers to ensure high linguistic accuracy of the spoken content. This manual transcription process was essential for capturing the nuances of the dialect, preserving emotion-related linguistic cues, and minimizing errors that may arise from automatic speech recognition systems. Table 1 Presents an example of the audio transcriptions across all emotion labels, showing both the original Moroccan Arabic script and the corresponding English translations.

**Table 1:** Examples of dataset structure showing Darija audio transcriptions and their corresponding English translations across emotion labels.

**Fig. 2:**
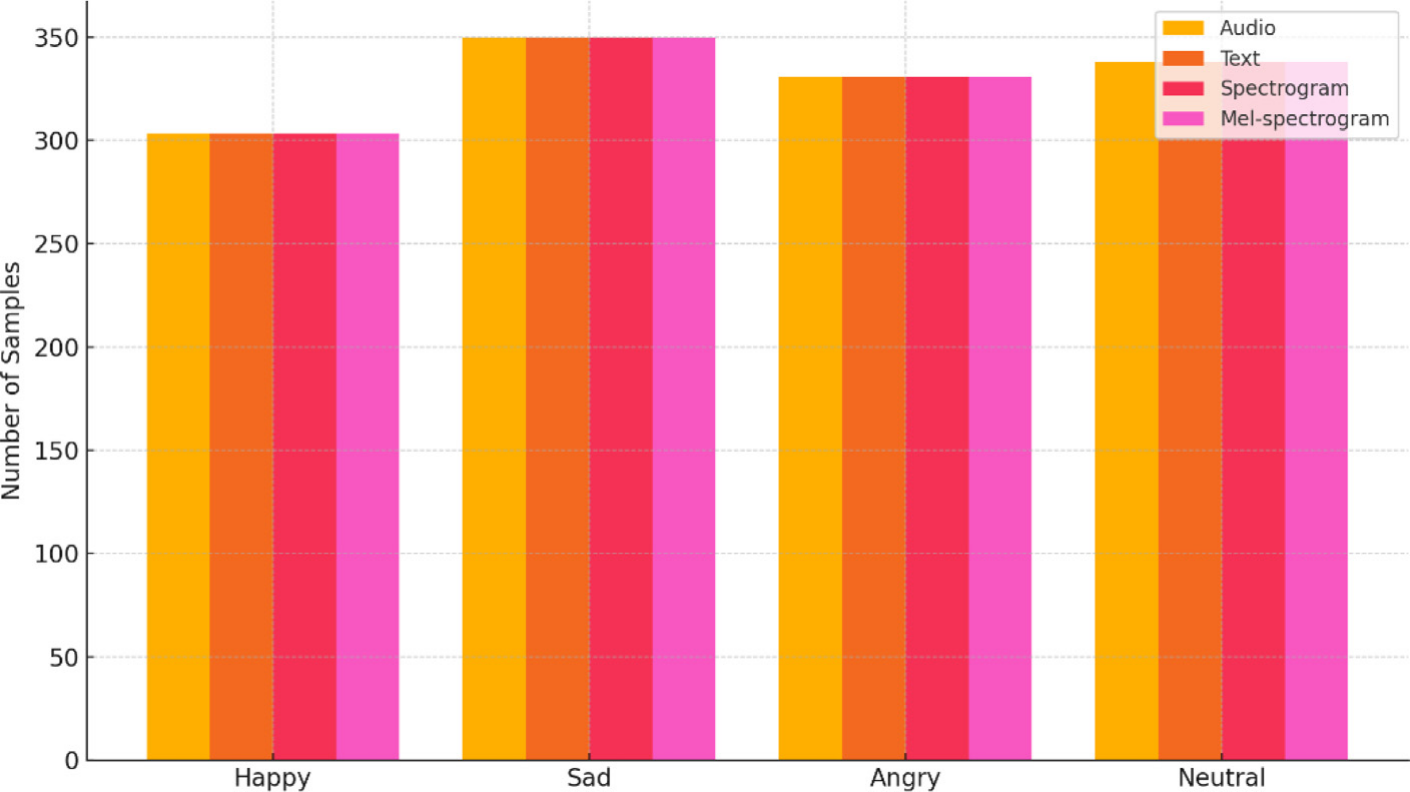
Distribution of samples per modality by emotion label.

**Fig. 3:**
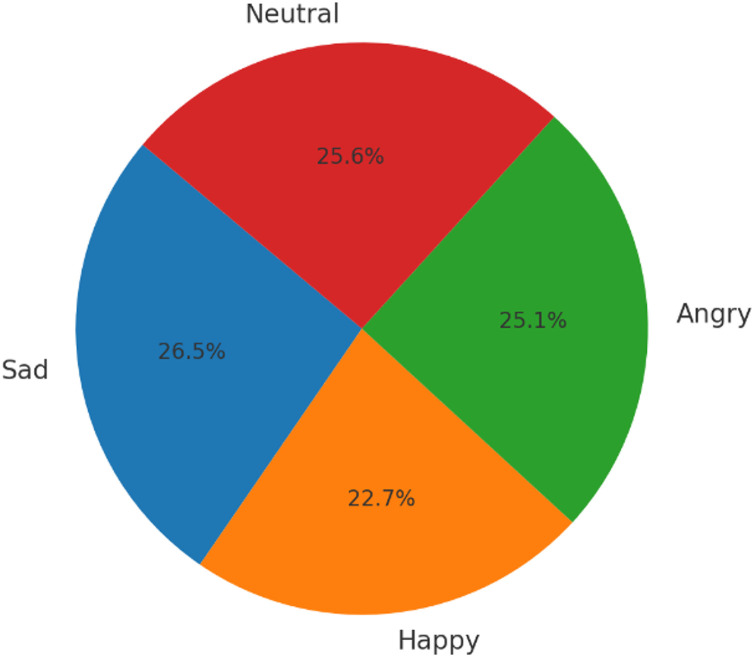
Emotion Distribution.

As illustrated in Fig. 2, Fig. 3 the number of samples per emotion label is approximately equal across all modalities. This uniform distribution contributes to the construction of a high-quality and balanced dataset, enabling its direct use in machine learning and deep learning tasks without the need for additional preprocessing or class-balancing techniques.

**Fig. 4:**
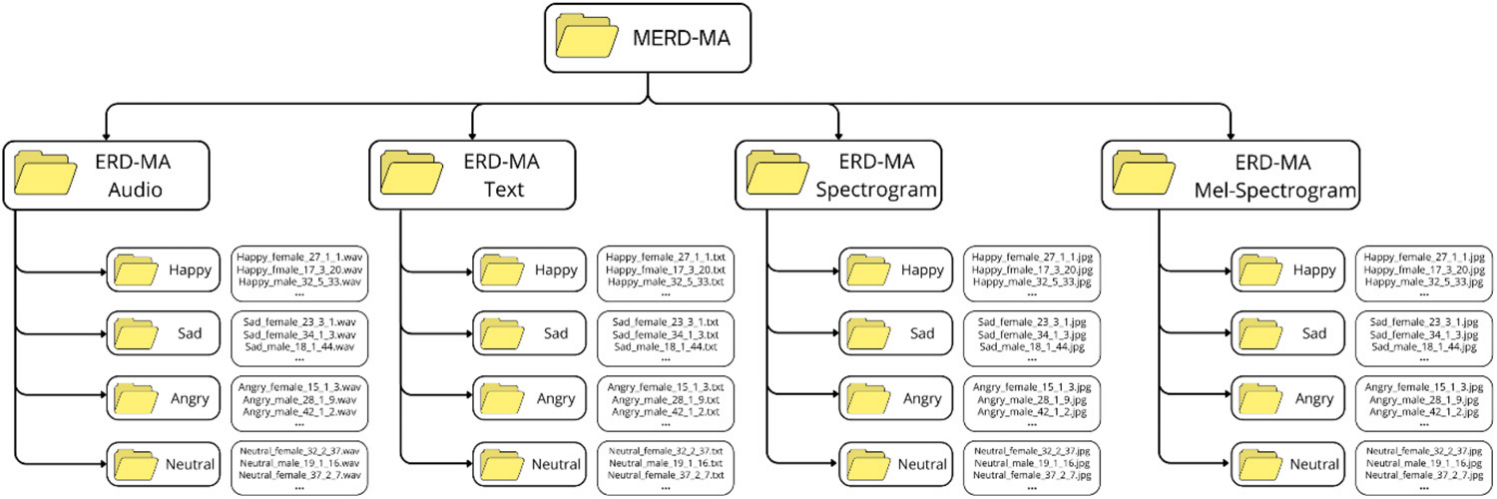
Structure of the MDER-MA dataset repository.

The dataset is freely accessible via https://data.mendeley.com/datasets/yzsw3ff6rn/1. As illustrated in Fig. 4 the repository is organized into four main directories, each corresponding to one data modality: ERD-MA Audio, ERD-MA Text, ERD-MA Spectrogram, and ERD-MA Mel-Spectrogram. Within each main folder, there are four subfolders labeled according to the target emotion categories: Happy, Sad, Angry, and Neutral. Each subfolder contains the samples associated with that specific emotion. This directory structure ensures that corresponding samples across all modalities can be easily aligned and retrieved, making the dataset straightforward to use in multimodal emotion recognition tasks.

To improve transparency and facilitate reuse, we provide a detailed data schema for the MDER-MA dataset, summarized in Table 2. The dataset includes multiple modalities, comprising audio recordings, text transcriptions, spectrograms, and mel-spectrogram images. Each audio file is stored in .wav format at 16 kHz, mono, and corresponds to a sentence-level transcription in Arabic script. Spectrograms and Mel-spectrograms images are generated from the audio and stored as image files in .jpg format, providing a visual representation of the speech signals for emotion analysis.

**Table 2:** Data schema of the MDER-MA emotion dataset.

File Type	Extension	Content	Level	Script	Additional Info
Audio	.wav	Speech recordings	Sentence-level	Arabic (Darija)	Mono, 16 kHz, 16-bit
Transcription	.txt	Text of spoken audio	Sentence-level	Arabic (Darija)	One sentence per line; corresponds to audio file name
Spectrogram	.jpg	Visual representation of audio	N/A	N/A	Generated from audio, size: 1000×400
Mel-Spectrogram	.jpg	Visual representation of audio	N/A	N/A	Generated from audio, size: 1000×400

**Fig. 5:**
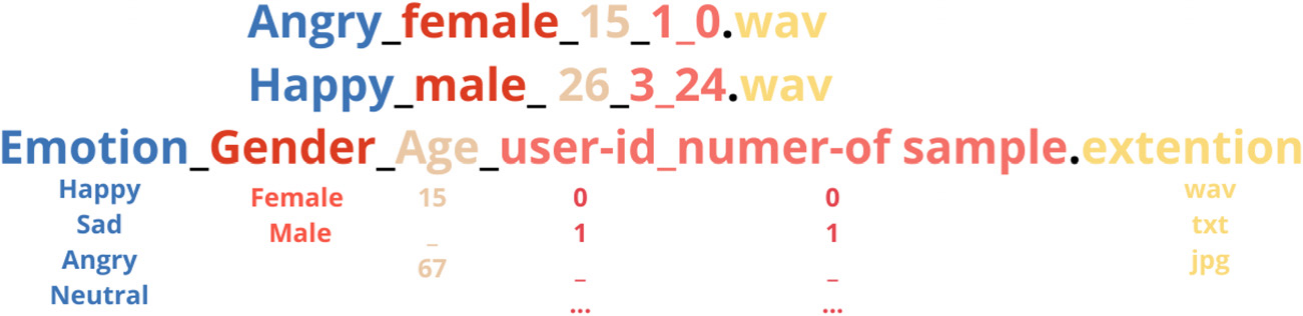
Dataset samples naming format.

To ensure consistency, traceability, and ease of data management across modalities, a structured naming was adopted for all files in the dataset. Each file name follows the format presented in Fig. 5. The Emotion field specifies the expressed emotion (e.g., Happy, Sad, Angry, Neutral), while Gender indicates the speaker's gender (male or female). Age refers to the speaker’s age, and UserID is a numeric identifier assigned to each speaker, allowing for tracking of multiple samples from the same individual. SampleNumber uniquely identifies each sample per speaker. The extension reflects the modality type: .wav for raw audio recordings, .txt for corresponding transcriptions, and .jpg for both spectrogram and Mel-spectrogram images. For instance, the file Angry_female_15_1_0.wav denotes an audio sample of an angry emotion spoken by a 15-year-old female speaker (user ID 1), and it is the first sample from that user. The same base naming is applied across all modalities, with only the file extension changing. This enables straightforward mapping and alignment of audio, transcription, and visual representations of the same emotional sample, thus facilitating efficient multimodal data integration and processing. Furthermore, as shown in Fig. 5, the file naming encodes four main attributes: emotion, gender, age of the speaker, and the user ID. This embedded metadata enhances the dataset’s versatility, enabling its use in additional tasks such as automatic age prediction and gender classification from Moroccan Arabic speech, alongside its primary application in emotion recognition.

The dataset comprises a total of 1322 audio samples collected from 83 unique participants (male and female). Each participant’s samples are identifiable via speaker IDs embedded in the filenames (e.g., *Angry_female_15_1_0* to *Angry_female_15_1_10* correspond to the same speaker). The clips range in duration from approximately 3 to 20 s, with no phrase repetitions across speakers. Because speaker identities are explicitly encoded, the dataset can be used for both speaker-dependent and speaker-independent emotion recognition tasks. However, given that all utterances are unique and not repeated across speakers, the dataset is more naturally suited to speaker-independent modeling.

**Fig. 6:**
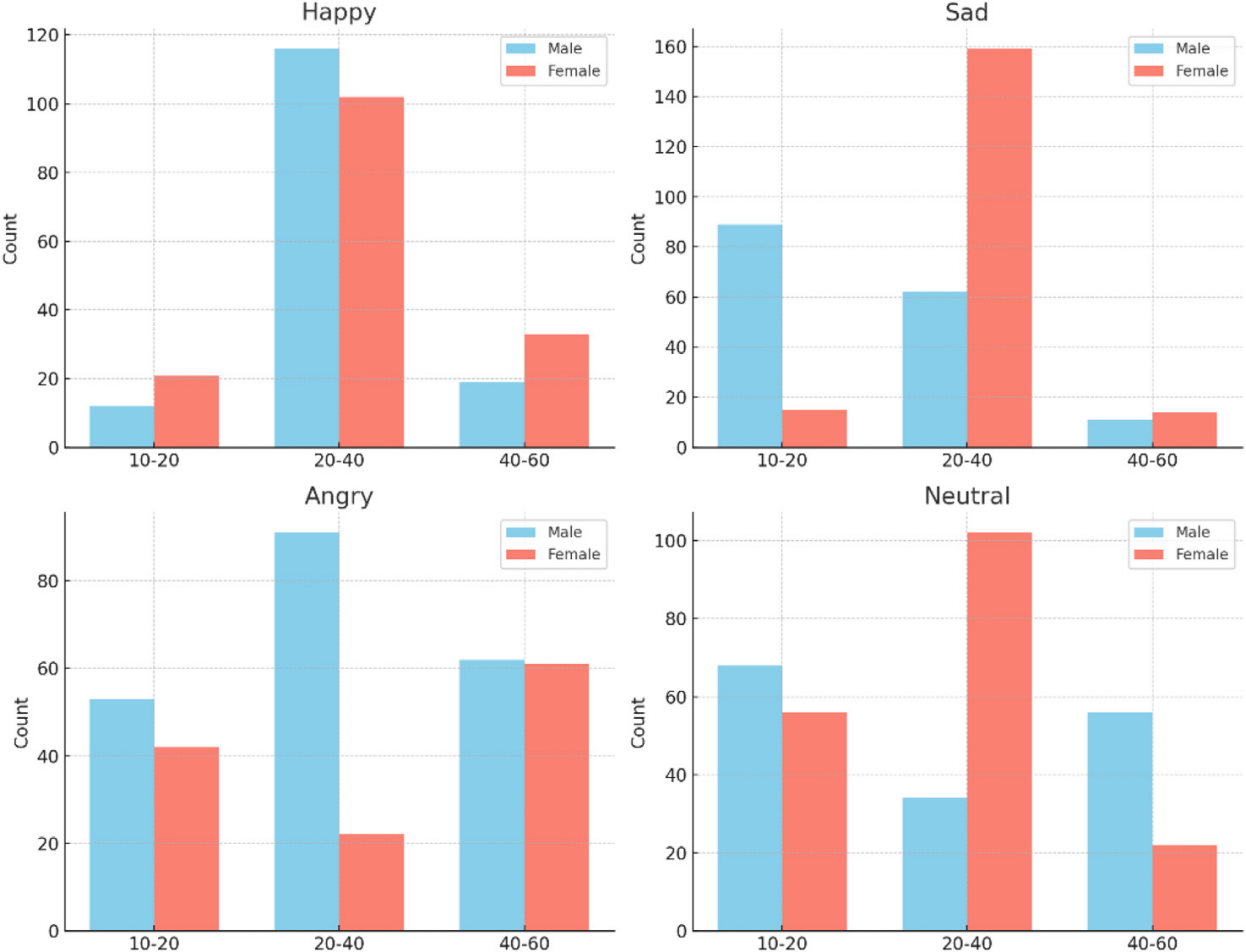
Distribution of emotions by Age and Gender.

**Fig. 7:**
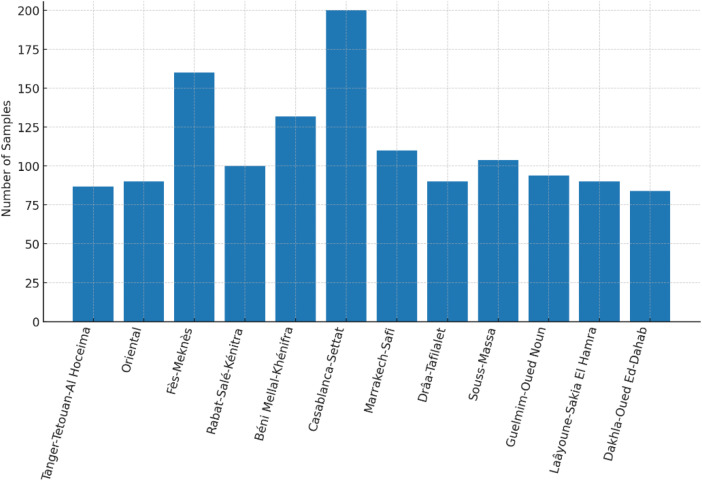
Dataset distribution of samples across regions.

Table 4 and Fig. 6 Presents the distribution of the dataset based on the age and gender of speakers. To ensure representativeness and diversity, audio samples were collected from both male and female speakers across a broad range of age groups. The dataset includes 673 samples from male speakers and 649 samples from female speakers, covering ages from 10 to 60 years old. By incorporating speakers from three distinct age brackets (10–20, 20–40, and 40–60), the dataset reflects a wide demographic spectrum, which makes it usable for other tasks such as age and gender classification from Moroccan speech. Moreover, since Morocco comprises 12 administrative regions [7], we aimed to avoid regional bias in our dataset, as emotional expression can vary significantly across different cultural and geographical contexts. To ensure broad coverage and enhance the representativeness of the dataset, we collected audio samples from all 12 regions of the country. As illustrated in Fig. 7 The distribution of samples across regions reflects our effort to include a diverse range of voices and dialectal variations, contributing to a more balanced and culturally inclusive resource for emotion recognition in Moroccan Arabic. However, it is important to note that all audio samples in the dataset are unique, and no phrases were repeated across speakers or modalities. As a result, explicit annotation of dialectal variation is not possible. Nevertheless, dialectal diversity is naturally embedded in the recordings through differences in vocabulary, pronunciation, and phonetic realization. To illustrate this implicit variation, Table 3 provides examples of transcription differences across three Moroccan regions. In total, the dataset contains 1322 unique utterances, reflecting both speaker and regional variability.

**Table 3:** Illustrative examples of Moroccan Arabic dialectal variation across regions.

**Table 4:** Dataset distribution based on age.

Emotion/ Gender	Male			Female			Total
	Age: [10–20]	Age: [20–40]	Age: [40–60]	Age: [10–20]	Age: [20–40]	Age: [40–60]	
Happy	12	116	19	21	102	33	303
Sad	89	62	11	15	159	14	350
Angry	53	91	62	42	22	61	331
Neutral	68	34	56	56	102	22	338
Total	222	303	148	134	385	130	1322

## 4 Experimental Design, Materials and Methods

**Fig. 8:**
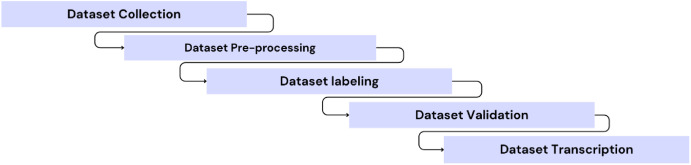
MDER-MA Dataset building pipeline.

As presented in Fig. 8, the construction of the MDER-MA dataset follows four main steps: data collection, pre-processing, labeling and validation, and data transcription. In this section, we provide a detailed explanation of each of these steps.

### 4.1 Dataset collection

MDER-MA audios were collected from diverse and authentic Moroccan media sources, including popular reality TV shows, radio programs, podcasts, and interviews. These sources were selected to reflect spontaneous and natural emotional expressions in real-life contexts. An initial pool of over 30 hours of audio content was gathered manually using Internet Download Manager (IDM). Special attention was given to speaker diversity, with an intentional balance of male and female voices and representation from all 12 administrative regions of Morocco. This geographic and demographic diversity was essential to building a culturally rich and regionally inclusive dataset. Since all data were obtained from publicly available online sources, no explicit consent was required for collection and use.

### 4.2 Dataset pre-processing

Following data collection, a thorough pre-processing phase was carried out to prepare the audio samples for annotation and analysis. The raw recordings were segmented into smaller clips using Audacity [8], an open-source audio editing software. Each segment was extracted based on clear emotional cues to ensure that every clip contained a single, distinct emotion. All audio files were converted and standardized into the .wav format. To protect speaker privacy and maintain ethical standards, any segments containing personally identifiable information, such as names, addresses, or sensitive references, were manually identified and removed. Additionally, as some recordings were sourced from noisy environments, a light noise reduction process was selectively applied using the built-in filtering features in Audacity. This process was carefully calibrated to preserve emotional content while improving audio clarity. Specifically, the noise reduction was applied with a reduction level of 10 dB, sensitivity of 2.00, and frequency smoothing of 4 bands. Only recordings with noticeable background noise were processed, ensuring that clean recordings remained untouched. This approach maintained both the high quality of the dataset and the integrity of emotional prosody, allowing the data to be suitable for emotion recognition research.

### 4.3 Dataset labeling and validation

To ensure the reliability of the dataset, a rigorous manual validation process was conducted by five native Moroccan Arabic speakers. Annotators were provided with detailed instructions defining the four target emotion categories (e.g., happy, sad, angry, and neutral) and were asked to assign each audio segment to the category that best reflected the speaker’s emotional state. If a segment did not correspond to any of the defined emotions, it was labeled as “none.” Each audio segment was independently reviewed by all annotators, and only samples that received the same emotion label from at least three annotators were retained in the final dataset. Disagreements were resolved using this majority-vote approach, which significantly reduced subjectivity and enhanced annotation consistency. Inter-annotator agreement was measured using Fleiss’ kappa, resulting in a value of **0.89**, indicating a perfect agreement. As a result, the validated dataset maintains a high degree of annotation quality, making it suitable for emotion recognition tasks*.*

### 4.4 Dataset transcription

The transcription of audio data was initially attempted using automatic speech recognition (ASR) tools, including Google Speech-to-Text and Whisper [9,10]. However, due to the limited support for Moroccan Darija by these systems, as well as the presence of minor background noise in some samples, the results were highly inaccurate and unreliable. Consequently, all transcriptions were conducted manually to ensure linguistic accuracy and cultural relevance. Each audio sample was transcribed in Darija using the Arabic script, reflecting the spoken content as naturally and authentically as possible. This manual approach, although time-consuming, was essential for preserving the nuances of the dialect and enabling accurate alignment with emotion labels. The resulting text data can be reliably used in text-based emotion recognition, speech-to-text benchmarking, and Arabic dialectal language modeling.

### 4.5 Benchmark experiment

To demonstrate the practical usability of the dataset, we conducted a benchmark text-based emotion classification experiment using DarijaBERT embeddings in combination with a Support Vector Machine (SVM) classifier. The transcriptions of all audio samples were first tokenized and processed by DarijaBERT to extract fixed-length embedding vectors representing the semantic content of each utterance. These embeddings were then used as input features to train a linear SVM classifier on an 80/20 train-test split to predict one of the four emotion categories: happy, sad, angry, or neutral. For the evaluation metrics, we used accuracy and F1-score. Using an SVM classifier on DarijaBERT embeddings, we achieved promising results, with an accuracy of 73 % and an F1-score of 71 %. Performance could be further improved by leveraging transformer-based models for end-to-end classification or by combining multiple modalities for decision-making. This baseline experiment demonstrates that the dataset is suitable for emotion recognition tasks and provides a reliable reference point for future research utilizing Moroccan Arabic speech and text data.

## Limitations

One of the main limitations encountered during the development of the MDER-MA dataset is the inherent complexity of accurately recognizing emotions in spontaneous speech. A fundamental challenge in emotion recognition lies in the non-binary and highly subjective nature of emotional expression. Audio samples with similar tone, pitch, or delivery style can convey different emotional meanings depending on various factors such as the context of the speech, the speaker's intention, and the cultural background of both the speaker and listener. To address this challenge, we implemented a rigorous validation process; however, minor bias may remain present.

## Ethics Statement

The authors confirm that they have read and followed the ethical requirements for publication in Data in Brief. This work does not involve human participants, animal experiments, or the use of data collected from social media platforms. All data used in this study were derived from publicly available, non-identifiable sources, and no personally identifiable information is present. The authors have anonymized all participant data and confirm that all the data is non-sensitive.

**Terms of service (ToS):** We are not using the data for any fraudulent activities such as making a profit (e.g., Business), DDoS, data theft, or any other sort of bad intention. Authors have considered and followed the source websites' ToS, privacy laws, and user consents.

**Copyright:** The data does NOT belong to users (e.g., social media). The data are published for free through user consent, and they can be consumed by anyone who has access to the Internet, so ethical approval has not been sought. We can confirm that this manuscript adheres to ethical publishing standards.

**Privacy:** Although the data is free and published in the public domain, we have anonymized all participant data and confirm that all the data is non-sensitive.

**Scraping policies:** There is no special scraping policy.

## CRediT authorship contribution statement

**Soufiyan Ouali:** Data curation, Methodology, Writing – original draft. **Said El Garouani:** Validation, Supervision, Writing – review & editing.

## Data Availability

Mendeley DataMDER-MA: Multimodal Emotion Recognition Dataset for the Moroccan Arabic (Original data).
